# Inflammatory Hibernoma of the Renal Hilum Mimicking a Renal Pelvis Tumor

**DOI:** 10.15586/jkcvhl.v9i1.225

**Published:** 2022-03-16

**Authors:** Kelsey Larkin, Kevin J. Chua, Sai Krishnaraya Doppalapudi, Colton Smith, Evita Sadimin, Valerie A. Fitzhugh, Eric A. Singer

**Affiliations:** 1 Section of Urologic Oncology, Rutgers Cancer Institute of New Jersey, New Brunswick, NJ, USA;; 2 Rutgers Robert Wood Johnson Medical School, New Brunswick, NJ, USA;; 3 Department of Pathology, Immunology and Laboratory Medicine, Rutgers Robert Wood Johnson Medical School, New Brunswick, NJ, USA;; 4 Section of Urologic Pathology, Rutgers Cancer Institute of New Jersey, New Brunswick, NJ, USA

**Keywords:** adipose tissue, brown, hibernoma, nephroureterectomy, renal pelvis, tumor

## Abstract

We report on an enhancing, heterogenous renal pelvis mass growing over 2 years which was found to be a benign hibernoma with inflammatory and lipomatous features originating from the renal hilum. To our knowledge, this is the first case reported on a hibernoma compressing on the renal pelvis and second case of a hibernoma with the inflammatory variant.

## Introduction

Hibernomas are rare tumors of brown adipose tissue and were named after similar tissue found in hibernating animals (1). Brown adipose tissue is abundant in newborns and hibernating animals, but its prevalence and metabolic activity in humans decline with progression of age. It primarily functions in thermoregulation when organisms need excess heat production (1). A phenomenon called nonshivering thermogenesis occurs with brown adipose, where heat generation occurs in the absence of muscular activity, mediated by the release of norepinephrine on increased exposure to cold temperatures (2).

While hibernomas are benign and have not shown potential for malignancy, they can present similarly to cancer as they grow over time and enhance on CT or MR imaging (2). We present a case of a renal pelvis mass concerning for upper tract urothelial cancer or renal cancer; however, instead an inflammatory hibernoma originating from the renal hilum was discovered. To our knowledge, this is the first case reported on a hibernoma compressing on the renal pelvis and second case of a hibernoma with the inflammatory variant.

## Case Report

A 77-year-old male with a history of benign prostatic hyperplasia and kidney stones had a renal ultrasound performed, which demonstrated mild left hydronephrosis. He subsequently underwent a CT and MRI revealing left hydronephrosis and a 2.6 × 2.9 cm left renal pelvis mass. Left retrograde pyelogram and left ureteroscopy demonstrated extrinsic compression of the renal pelvis, and biopsy and cytology were negative for malignancy. MDM2 fluorescence in situ hybridization (FISH) was negative for amplification. The patient was subsequently referred to our institution for further evaluation of the mass.

**Figure 1:**
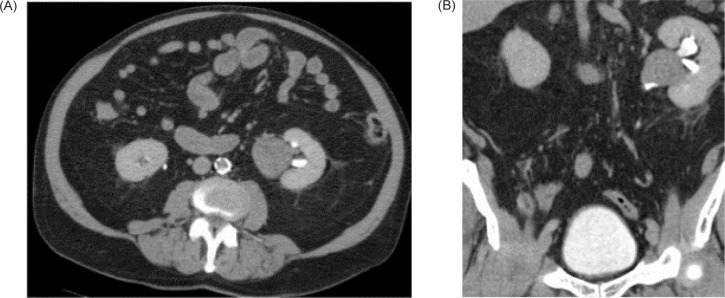
Left renal pelvis mass on delayed phase of CT Urogram. (A) Axial (B) Coronal

An initial fine needle aspiration (FNA) of the left renal pelvis mass detected inflammatory infiltrate and adipocytes without cancer. Subsequent CT urography demonstrated that the lesion was slightly enhancing and had grown to 3.3 × 3.2 cm. Given the patient’s chronic renal disease, and the absence of malignant cells seen on urine cytology and percutaneous biopsy, surveillance was selected. Repeated CT urography showed interval increase in size to 3.7 × 3.5 cm, and follow-up MRI demonstrated that the mass had solid-enhancing components consistent with a neoplasm. However, repeated percutaneous biopsy (core needle and FNA) demonstrated an inflammatory infiltrate with adipocytes. MDM2 FISH was again negative for amplification. Diethylenetriamine pentaacetate (DTPA) renal scan with furosemide performed and then repeated a year and a half later revealed left kidney functioning at 45 and 46%, respectively, and right kidney functioning at 55 and 54%, respectively, without definitive evidence of high-grade obstruction. A follow-up CT urography showed the complex heterogenous left renal pelvis mass had increased in size to 4.0 × 3.4 cm with enhancement (Figure 1).

Due to concern of malignancy, given the growing, enhancing, and complex cystic lesion with solid components, patient elected to undergo left robotic nephroureterectomy with retroperitoneal lymph node dissection.

**Figure 2:**
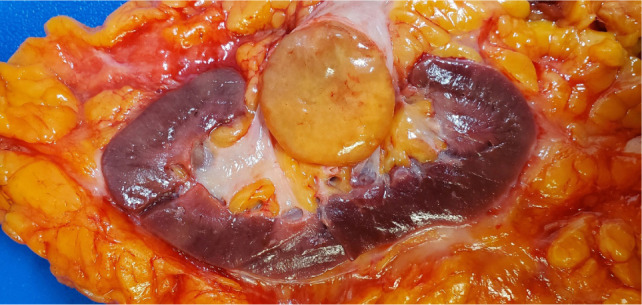
Gross resection specimen of the left renal hibernoma.

**Figure 3:**
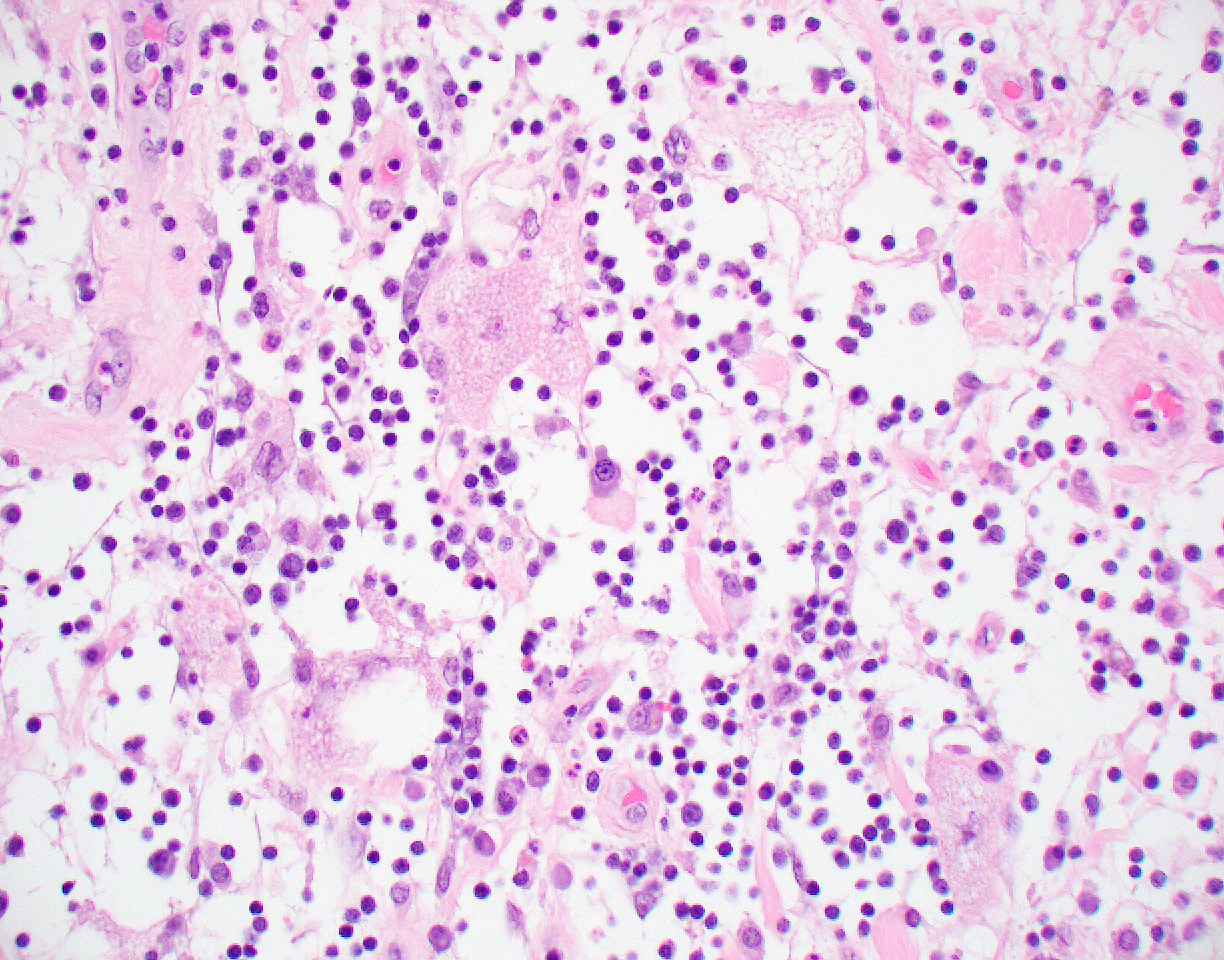
High-power histologic photomicrograph demonstrating scattered brown adipocytes invested by an extensive lymphoplasmacytic inflammatory cell infiltrate (hematoxylin and eosin, 40X).

Upon gross examination, the lesion measured 4.0 × 3.5 × 3.5 cm with a pale white capsule located at the hilum and pushing inwards to the renal pelvis. The lesion was yellow or tan and well circumscribed (Figure 2). Twenty-four para-aortic lymph nodes were removed, and all were benign. Microscopic examination revealed adipose tissue with the tissue closest to the capsule containing the highest density of inflammatory cells. Scattered brown and white adipocytes became more prominent toward the center of the lesion. An example of brown adipocytes surrounded by extensive inflammation is demonstrated in Figure 3. No definitive lipoblasts were identified. The brown adipocytes had diffuse immunoreactivity to S-100 and were negative for MART1, HMB45, smooth muscle actin, and cathepsin. S-100 has been shown to have at least focal positivity in most hibernomas (1). The differential diagnosis of inflammatory liposarcoma was excluded because of the lack of MDM2 amplification via FISH in three separate specimens, two from the prior biopsy and one from the resection specimen.

## Discussion

Hibernomas are tumors of brown adipose which most commonly occur in the thigh, shoulder, neck, and back, and less commonly seen in the abdominal cavity and retroperitoneum (1). Reports of hibernomas found around the ureter and kidney are rare (1–3). Interestingly, we report on a case of a growing and enhancing lesion at the left renal pelvis which was found to be an inflammatory hibernoma.

Hibernomas have various morphological variants, including typical, spindle, lipoma-like, myxoid, and inflammatory (3). The typical variant is defined by a mixture of multivacuolated brown fat cells and univacuolated mature adipocytes. The lipoma-like variant, partially identified in our case, consists of mainly mature adipocytes with rarer brown adipocytes. The myxoid variant contains hibernoma cells dispersed throughout an acellular myxoid stroma. The spindle cell variant has hibernoma cells dispersed in a myxoid stroma consisting of dense collagen, mast cells, and bland spindled cells (3). The inflammatory type, only previously described once by Streich and Yang and reported in our case, demonstrates a hibernoma with a dense lymphoplasmacytic infiltrate.

Diagnosis of a renal hibernoma can be difficult given that it can present as a growing, enhancing heterogeneous mass on cross-sectional imaging. Percutaneous biopsies can be considered; however, a negative biopsy is not definitive because renal mass biopsies are nondiagnostic about 14% of the time (4). Additionally, there is a risk of bleeding given a hibernoma’s vascularity (2). Lemos et al. identified unique cytologic features of hibernomas which can help rule out the diagnosis of a liposarcoma during an FNA. However, they also acknowledged the difficulties in distinguishing types of fatty lesions as their features appear very similar (5). Surgical excision is the diagnostic and treatment method of choice for identifying hibernomas as they are indistinguishable from malignancy clinically and radiographically.

## Conclusion

We report on a rare case of a renal pelvis lesion found to be a benign hibernoma and the second known report of a hibernoma with the inflammatory variant. It is important to consider hibernomas on the differential for renal and upper urinary tract masses. Surgical excision is necessary for diagnosis and treatment as they are often clinically and radiographically indistinguishable from a malignancy.
